# Smart DNA sensors‐based molecular identification for cancer subtyping

**DOI:** 10.1002/smo.20230020

**Published:** 2023-12-12

**Authors:** Yingying Gao, Mengyi Xiong, Chaonan Gong, Bo Wang, Longwei Bai, Xiao‐Bing Zhang

**Affiliations:** ^1^ Molecular Science and Biomedicine Laboratory State Key Laboratory of Chemo/Biosensing and Chemometrics Collaborative Innovation Center for Chemistry and Molecular Medicine College of Chemistry and Chemical Engineering Hunan University Changsha Hunan China

**Keywords:** biomarkers, DNA sensors, logical computing, molecular subtyping

## Abstract

Molecular subtyping of cancer can greatly help to understand the development of disease and predict tumor behavior. Exploring detection methods for precise subtyping is appealing to prognosis and personalized therapy. During the past decades, DNA‐based biosensors have exhibited great potential in cancer diagnosis due to their structural programmability and functional diversity. Despite the encouraging progress that has been made, there remains an issue in improving the accuracy and sensitivity of cancer subtyping due to the complex process of disease, especially in preclinical or clinical applications. To accelerate the development of DNA sensors in the identification of cancer subtypes, in this review, we summarized their advances in molecular subtyping by analyzing the heterogeneity in categories and levels of biomarkers between cancer subtypes. The strategies toward genomic and proteomic heterogeneity in cells or on the cell surface, as well as the cancer excretions including extracellular vesicles (EVs) and microRNA (miRNAs) in serum, are summarized. Current challenges and the opportunities of DNA‐based sensors in this field are also discussed.

## Graphical abstract

Molecular subtyping of cancer is crucial for understanding the development of disease and predicting tumor behavior. This review focuses on the advanced strategies of molecular subtyping using DNA‐based biosensors for the identification of cancer subtypes based on different biomarkers, including cells, extracellular vesicles (EVs), and microRNAs (miRNAs) in serum.

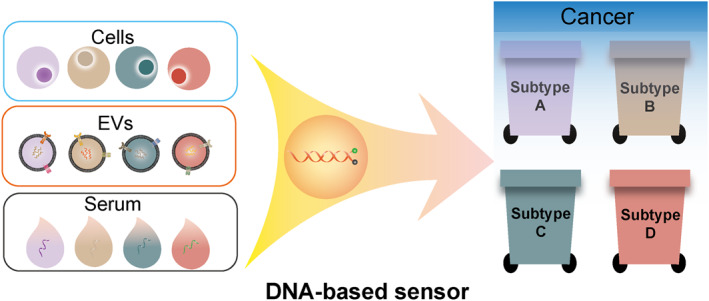

## 1 INTRODUCTION

The subtypes of cancer that exhibit morphological similarity often have dramatically different features, function variably in the disease's development, and respond inconsistently to therapy.^[1]^ Clinical evidence has demonstrated that differences in the molecular pathology of indistinguishable cancer subtypes substantially impact disease characteristics.[^1^, ^2^] Therefore, to improve the therapeutic effect and reduce treatment expense, it is important to formulate appropriate therapeutic strategies for cancer subtypes before treatment. However, current clinical diagnosis usually relies on histopathological criteria for cancerous subtyping, which fails to comprehensively reflect the true heterogenic character of cancer. Advanced diagnostic technologies have revealed that the histopathological heterogeneity of tumors usually results from substantial molecular differences.^[3]^ Molecular subtyping studies have allowed the classification of cancer subtypes into uniform groups that correlate better with clinical outcomes than the traditional cancer classifications.

Cancer is substantially a kind of genetic disease in which the mutations at the single‐nucleotide level or much larger scales induce genomic heterogeneity.^[4]^ Based on the histological appearance and site of growth, the traditional classification of cancer only partially captures the true heterogenic character of cancer. Recent advances in genome‐wide profiling techniques have largely accelerated the development of cancer subtyping studies. Derived from genomic mutations, the heterogeneity can be transferred downstream to the transcriptome, then to the proteome, and is finally displayed as the differences in the metabolome.[^4^, ^5^] As a result, these heterogeneities provide many biomarkers for identifying cancer subtypes, ranging from genes, and proteins to metabolic molecules.[^3^, ^6^] By analyzing these biomarkers in clinical samples, researchers can distinguish the subtypes of cancers. Therefore, exploring analytical methods toward these molecular markers can accelerate the advancement of the subtyping of cancer.

Recently, DNA sensors with increasing structural and functional complexity have been extensively used in the classification of cancer subtypes due to several features.^[7]^ First, DNA possesses a unique instinct of hybridizing with its complementary sequences that allows the detection of genic targets.^[8]^ Beyond this nature, DNA can also recognize a wide range of targets. The most typical example is DNA aptamers, a class of antibody‐like nucleic acid ligands screened through in vitro selection.^[9]^ DNA aptamers that can bind various targets have been discovered, including metal ions, small molecules, proteins, peptides, bacteria, viruses, and cells.^[10]^ Another obvious advantage of DNA sensors is their talent in signal amplification. Either the enzyme‐assisted polymerization, such as polymerase chain reaction (PCR)^[11]^ and rolling circle amplification (RCA),^[12]^ or the toehold‐mediated strand displacement (TMSD) including hybridization chain reaction (HCR)^[13]^ and catalytic hairpin assembly (CHA),^[14]^ has exhibited superior capability in signal amplification, which allows the assays of trace targets of sub‐picomole or lower. Finally, the interactions and thermodynamics of DNA hybridizations are highly predictable.^[15]^ Based on these natural features, DNA can recognize different molecules, process input information, and output result signals in a programmable manner, which is called DNA logical computing.

Given these features, DNA sensors exhibit great potential in the identification of cancer subtypes by analyzing not only the intracellular or surface biomarkers between them but also the metabolite secreted by cells. Based on dynamic DNA nanotechnology, strategies such as DNA‐based computing network reactions and in situ triggered DNA amplification reactions have been utilized for cancer subtyping. Compared to traditional classification of cancer such as immunohistochemistry (IHC), DNA sensors are the better methods in reflecting the individual patient's biological information and enriching the toolbox of molecular subtyping. To further accelerate the progress of DNA sensors and promote their preclinical or clinical applications, herein, we intend to summarize the latest advancements in DNA sensors in cancer subtyping (Table 1). First, we discuss the strategies that analyze the heterogeneity in categories and levels of cellular contents such as membrane proteins and the microRNA/mRNA/DNA or both between cancer subtypes. Then, the heterogeneity in cellular secretions, especially in serum samples, including extracellular vesicles and miRNA, is also investigated. Finally, current challenges and their potential solutions are discussed in cancer subtyping, as well as the opportunities.

**TABLE 1:** Summary of DNA sensors for cancer subtyping.

Origins	Target	Signal amplification	Recognition method	Disease	Signal output	Clinical samples	Accuracy	Required time	Reference
Cells	miRNA	TMSD	Complementary DNA (cDNA）	Breast cancer	Fluorescence	No	N.A.	N.A.	[23]
	miRNA	DNAzyme	cDNA	Breast cancer	Fluorescence	Yes	N.A.	4 h	[24]
	miRNA	CHA	cDNA	Breast cancer	Mass spectrometry	No	N.A.	4 h	[25]
	miRNA	T7 exonuclease	cDNA	Breast cancer	Electrochemistry	No	N.A.	N.A.	[26]
	Cell receptor	No	Aptamer	Leukemia	Fluorescence	No	N.A.	N.A.	[30, 31]
	Cell receptor	HCR	Aptamer	Leukemia	Fluorescence	No	N.A.	>1 h	[32]
	Cell receptor	RCA	Aptamer	Leukemia	Fluorescence	Yes	N.A.	N.A.	[33]
	Cell receptor	CRISPR cas12a	Aptamer	Leukemia	Fluorescence	No	N.A.	20 min	[34]
	Cell receptor	No	Aptamer	Different cancer	Fluorescence	No	N.A.	>1 h	[35, 36]
	Cell receptor and miRNA	TMSD	Aptamer and cDNA	Different cancer	Fluorescence	No	N.A.	N.A.	[37]
	Cell receptor	Nicking endonuclease	Antibody	Different cancer	Fluorescence	No	N.A.	N.A.	[38]
	Cell receptor	TMSD	Antibody	Breast cancer	Electrochemical	No	N.A.	120 min	[40]
Exosome	miRNA	Thermophoresis	cDNA	Breast cancer	Fluorescence	Yes	Over 85%	N.A.	[44, 45]
	Cell receptor	HCR and Thermophoresis	Aptamer	Breast cancer	Fluorescence	Yes	97%	N.A.	[46]
	miRNA	TMSD	Homotypic	Breast cancer	Electrochemistry	Yes	N.A.	N.A.	[47]
Serum	miRNAs	PCR	cDNA	NSCLC	Fluorescence	Yes	86.40%	6 h	[49]
	mRNA	PCR	cDNA	ARI	Fluorescence	Yes	87%	4 h	[50]
	Genome	PCR	cDNA	CYP2C19	Fluorescence	No	Similar to Sanger sequencing	N.A.	[51]

## 2 CELLULAR BIOMARKER FOR DIFFERENTIATING SUBTYPES OF CANCER BASED ON CELLS

Abnormal gene or protein expression usually exists in the occurrence and development of cancer, which can act as a promising biomarker for cancer diagnosis.^[16]^ However, normal cells possess most biomarkers at a low level that is overexpressed in cancerous cells. The single‐biomarker strategy of discrimination for cell subtyping may result in off‐target effects and unsatisfactory information about the pathological processes.^[17]^ Accordingly, for the subpopulation of cancer cells that have no characteristic markers, it is necessary to develop a strategy of accurate identification based on multiple markers.

*Intracellular biomarkers for discrimination*. Genetic heterogeneity in cells is a reliable rationale for cell identification. Increasing evidence suggests that different cancers express RNA molecules inconsistently, which can act as biomarkers.^[18]^ Particularly microRNA (miRNAs), a family of endogenous noncoding RNA molecules with 19–23 nucleotides (nt), can manipulate gene expression by regulating mRNA translation.^[19]^ Dysregulated miRNA is correlated with the prognosis of various diseases, even cancers. Consequently, intracellular miRNA profiling could be instrumental in earlier cancer diagnosis as non‐invasive biomarkers. Nevertheless, the expression of miRNAs is highly cell type‐specific, low abundant, and dynamic.^[20]^ Thus, numerous strategies have been developed for sensitive in situ monitoring of intracellular miRNAs sensitively.^[21]^ In addition, considering the role of miRNA expression levels in classifying cell subtypes, reliable methods for the one‐time detection of several miRNAs were developed for the precise discrimination of cancer subtypes.

**FIGURE 1:**
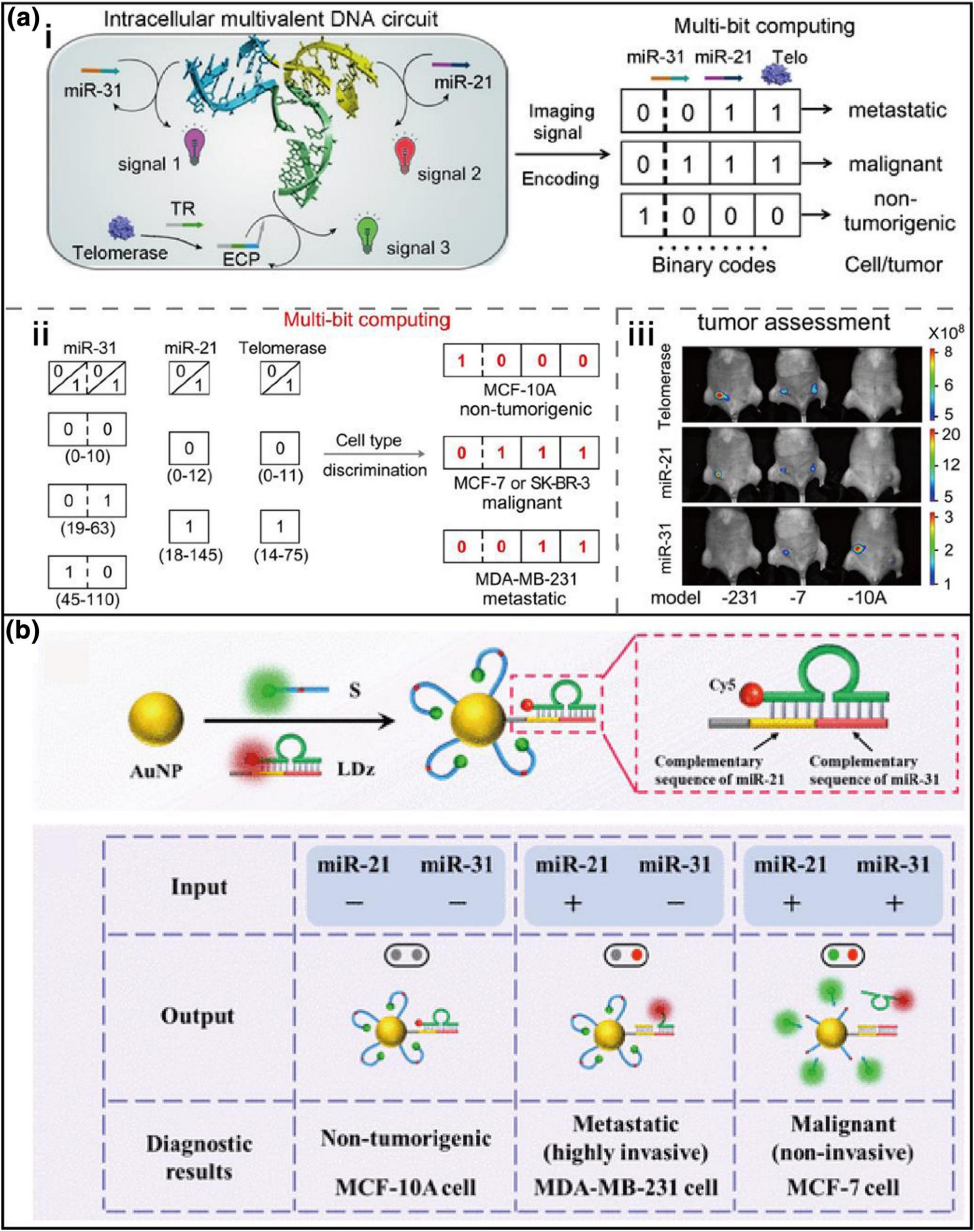
Intracellular miRNAs for discrimination of cancer cell subtypes. (a) DNA recycling and multi‐bit computing process of the multivalent DNA nanodevice for subtyping of three breast cancer subtypes.^[23]^ (b) The dual‐miRNA‐responsive three‐dimensional DNA nanomachine for the subtype differentiation of breast cancer cells.^[24]^ Graph copyright from reference [^23^, ^24^].

Dynamic DNA circuits, based on programmable DNA hybridization and strand displacement processes, have been widely employed to perform intelligent computation, such as sensing and imaging.^[22]^ To this end, DNA sensors are powerful tools for promising information in the discrimination of cancer subtypes. For example, Zhao's group^[23]^ developed an entropy‐driven DNA nanodevice for the subtyping of breast cancer cell types (Figure 1a). In this design, telomerase catalysis product and miRNAs (miR21 and miR31) could trigger the TMSD reactions, for which these two miRNAs and telomerase together were transduced into multi‐bit binary codes, allowing the discrimination of MCF‐10A (non‐tumorigenic), MCF‐7 (malignant) and MDA‐MB‐231 (metastatic) breast cells (Figure 1b,c). The imaging signals of this method were encoded as multi‐bit binary codes and used for the first time to discriminate the subtypes of breast cancer as well as respective tumors. Though these DNA reactions perform well in test tubes, their applications in living cells are difficult. Because the highly negative charge of DNA hinders the direct cellular uptake of DNA sensors. Therefore, some nanomaterials are employed as carriers to deliver DNA sensor components into living cells. Li's group^[24]^ constructed a dual‐miRNA‐responsive three‐dimensional DNA walker employing gold nanoparticles for the differentiation of breast cancer cell subtypes (Figure 1d). By triggering with endogenous miR21 and miR31, DNAzyme was activated to cleave substrate strands conjugated to the AuNPs for signal amplification. This strategy exhibited superior sensitivity and selectivity, realizing the cell subtypes discrimination accurately of breast cancer.

**FIGURE 2:**
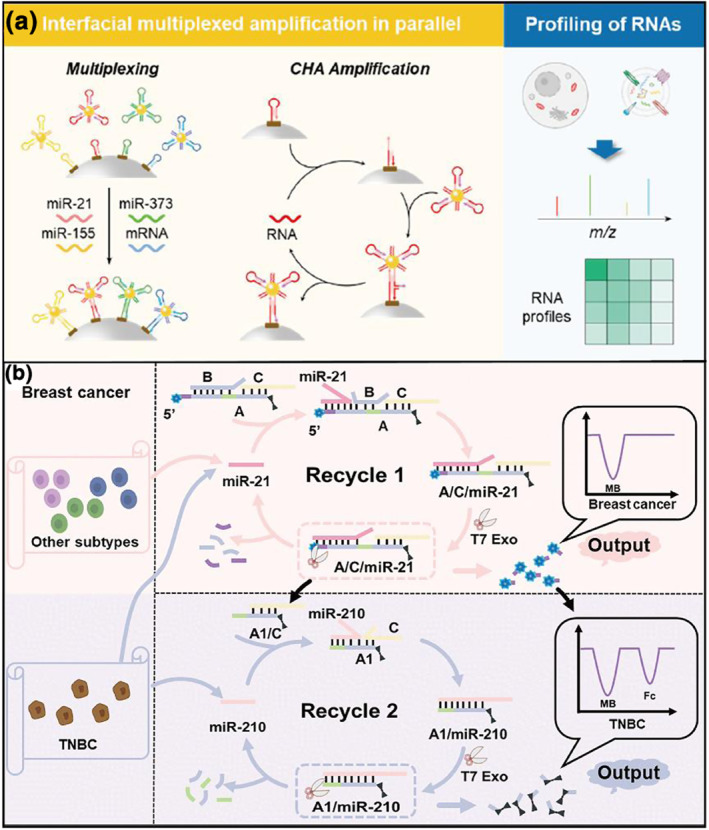
DNA sensors with different signal outputs for intracellular miRNA sensing. (a) MALDI‐MS for simultaneously detecting multiple miRNA biomarkers from the subtypes of different breast cancers.^[25]^ (b) Enzyme‐driven miRNA detection with the electrochemical measurement in the subtype profiling of breast cancer cells.^[26]^ Graph copyright from reference [^25^, ^26^].

Current DNA sensors usually use fluorescence as signal readout, which may hinder the assay multiplex targets because of the spectral overlap of fluorophores. To meet this challenge, Min et al^[25]^ presented a simultaneous assay of multiple RNAs by matrix‐assisted laser desorption/ionization mass spectrometry (MALDI‐MS) (Figure 2a). Different from fluorescence spectrometry, mass spectrometry (MS) distinguishes multiple targets by the characteristic ion signal, thereby providing a broad spectral window for multiplexed analysis. By conducting a CHA reaction on nanointerfaces, the encoding RNAs cyclically assembled on magnetic beads can generate multiplexed and amplified characteristic ion signals corresponding to target RNAs upon MALDI‐MS, allowing precision subtyping of diverse breast cancer cell subtypes. However, this method is time‐consuming and exhibits limits of detection at the sub‐pM level. Alternatively, Zhao's group^[26]^ reported enzyme‐assisted DNA signal amplification for cancer subtyping based on an electrochemical technique (Figure 2b). The miRNA discriminator was involved in two TMSD reactions, in which two miRNA biomarkers (miR21 and miR210) initiated strand displacement sequentially and a T7 exonuclease (EXO) powered digestion procedure. With an electrochemical output signal, this method successfully distinguished breast cancer cells from normal cells and even identified triple‐negative cells, which achieved a high sensitivity of fM levels for the miRNA biomarkers. The MS and electrochemical methods extend the output signals toolbox of DNA sensors in the subtyping of cancer.

*Cell membrane receptors for discrimination*. Cell‐surface receptors play a vital role in the biological reactions and communication between cells.^[27]^ These receptors can identify external stimuli and prompt intracellular signaling such as proliferation, migration, and differentiation. In particular, the abnormal expression of membrane receptors may cause dysfunctional cellular metabolism, signaling transduction, and proliferation in cancer cells.^[28]^ By analyzing the differences in the expression levels of multiple membrane biomarkers, diseased cells can be precisely targeted and identified. However, the subpopulations of cancer cells and even normal cells may overexpress identical molecular markers, for which the identification of specific subtypes of cancer cells through a single biomarker is regarded as incredible.^[29]^ Therefore, simultaneous identification of multiple membrane markers is more practical and effective for accurate profiling and quick analysis of cancer subtypes.

**FIGURE 3:**
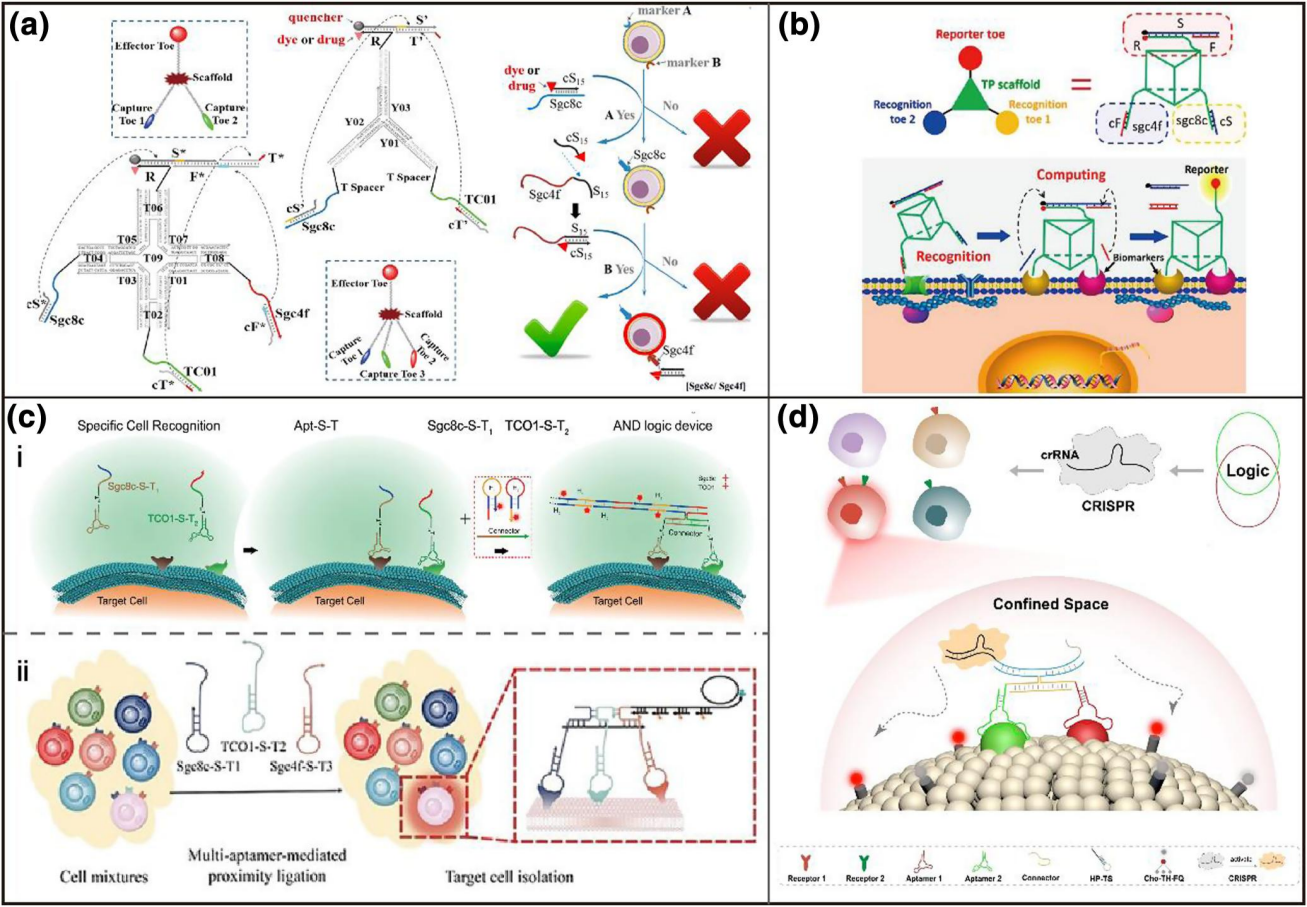
Cell membrane receptors for the discrimination of cancer cell subtypes. (a) Designs of trivalent “Y”‐shaped Nano‐Claw for two inputs and tetravalent “X”‐shaped Nano‐Claw for three inputs in the analysis of multiple cancer membrane biomarkers.^[30]^ (b) Working principles of DNA‐engineered nanomachine for better molecular targeting.^[31]^ (c) System design and operational mechanism based on HCR^[32]^ and RCA^[33]^ for accurate cancer cell identification. (d) Illustration of fluidly confined CRISPR‐based DNA reporter (FINDER) for cancer cell identification.^[34]^ Graph copyright from reference [^30^, ^31^, ^32^, ^33^, ^34^].

In traditional methods, antibodies can bind surface receptors that are more highly expressed in tumors, which can be utilized to diagnose disease between analogous cancers. Similarly, DNA aptamers, a class of short DNA strands, can also bind various targets ranging from small molecules to proteins.^[10c]^ Many aptamers have been demonstrated to profile the differences in the expression levels of cellular receptors between many cell subtypes. To further improve the accuracy, aptamer/antibody‐engaged DNA circuit has been a booming approach to distinguish from cell subtypes with high specificity, programmability, and biocomputational ability. You et al^[30]^ designed a “Nano‐Claw” that used three aptamers as building blocks for target cells with three different receptors expressed (Figure 3a). This logic robot consists of “capture toes” and “effector toe” which are constructed by several structure‐switchable aptamers and a logic‐gated DNA duplex, respectively. The logic sensors can recognize multiple targets as inputs and isolate target cells with high specificity. However, these methods based on the molecular proximity effect all comprise several components that are freely diffusible in a reaction buffer. Thus, the logic recognition speed is severely limited. Therefore, Peng et al^[31]^ developed an aptamer‐based DNA nanomachine for target cell membrane computing (Figure 3b). Two aptamers were selected to recognize the overexpressed cell receptors on human acute lymphoblastic leukemia cells. Once binding to the target cells, two trigger strands were released to drive the DNA strand displacement reactions and presented an “on” signal. Though these logic DNA devices could distinguish the subtypes of cancer cells, the linear signal output in the multiple steps of logic computations might result in low sensitivity. To achieve high specificity and sensitivity, DNA catalytic reactions have been used for in situ signal amplification on the membrane receptors, such as HCR^[32]^ and CHA^[33]^ (Figure 3c). By integrating the recognition of multiple aptamers with DNA signal amplification strategies, an AND logic analysis pattern was successfully operated on the cell surface. These approaches can precisely label the target cell to distinguish the subtypes based on the expression level of different biomarkers. These methods exhibited encouraging efficacy in differentiating tumor cell subtypes in cell mixtures, even in clinical specimens. Nevertheless, this method still requires at least 1 h detection time. Recently, to realize more rapid and sensitive identification for target cells, our group developed a CRISPR‐based DNA reporter based on fluidly confined cell membranes named FINDER, in which an aptamer‐based DNA logic gate was used for identifying the target cell and CRISPR Cas12a ‐based was used for the signal output (Figure 3d).^[34]^ The activity of CRISPR nuclease was significantly enhanced on the cell membrane due to the confinement effect and membrane fluidity. Finally, the FINDER could identify 0.1% of target cancer cells with over 80% recognition efficiency in 20 min. This method exhibited a rapid and sensitive method for the identification of cancer cells, thereby indicating its potential application in personalized medicine and biomedical engineering.

**FIGURE 4:**
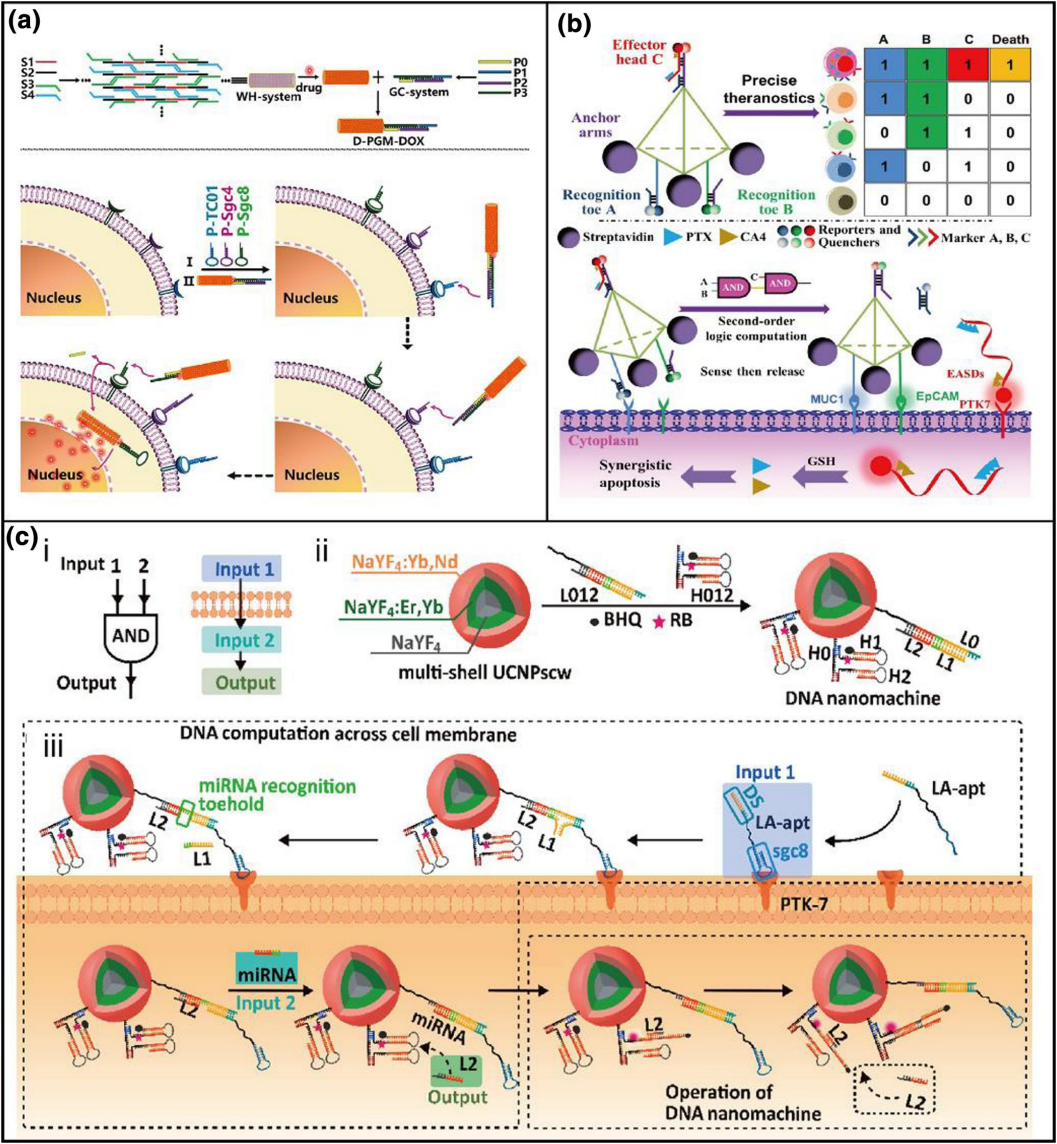
DNA nanosensors with cancer cell identification and target therapy. (a) Illustration of D‐PGM for cancer cell‐targeted identification and therapy based on three receptors recognition.^[35]^ (b) Molecular design and operational design of DNA logic‐guided nanorobot on cell membrane for multiplexed diagnosing and synergistic killing.^[36]^ (c) The transmembrane DNA computation strategy for precise therapy for solid tumors.^[37]^ Graph copyright from reference [^35^, ^36^, ^37^].

Additionally, it is precise and efficient to utilize DNA nanosensors to deliver drugs to target cells. Accordingly, Wu^[35]^ et al developed a DNA missile consisting of a warhead (WH) and guidance/control (GC) called D‐PGM (Figure 4a). The GC was a smaller DNA logic circuit construction of three different aptamers, and WH was a three‐dimensional DNA nanostructure that served as a drug carrier compartment. The target cell membrane co‐expressed biomarkers were recognized and disassembled by Sgc8, Sgc4f, and TC01 logically, and sequentially fully released DOX toward target cells even when exposed to similar cells. To further expand the multiplexed‐diagnose capability of DNA nanosensors on the cell membrane, Tan's group^[36]^ developed a second‐order DNA logic‐gated nanorobot with multiple aptamers that anchor on the cell membrane for multiplexed diagnosing and synergistic killing simultaneously (Figure 4b). For this second‐order nanostructure, the binding of aptamers with three markers (PTK7, MUC1, and EpCAM) on the target cells would release two blocker strands. Finally, fluorescence or synergistic drugs are internalized to realize the diagnosis and targeted therapy. Apart from DNA nanosensors only at the cellular outward or inward cell membrane, a transmembrane DNA nanomachine with a logical computation function was developed to distinguish them from the complex tumor microenvironment. Ju et al^[37]^ utilized an sgc8 of LA‐apt targeting for PTK‐7, and then the DS of LA‐apt captured and reacted with the DNA nanomachine (Figure 4c). With the assistance of PTK‐7, the DNA nanomachine endocytosis into the cancer cell and then reacted with miRNA‐21. This method depends on two‐biomarker activation for the precise recognition of tumors, which is a promising approach to target cancer cells. All the examples demonstrate that DNA logic circuits can accurately identify target cancer cells in a complex biological milieu and target cancer therapy in a smart method, further demonstrating the importance of DNA sensors in cancer subtyping.

**FIGURE 5:**
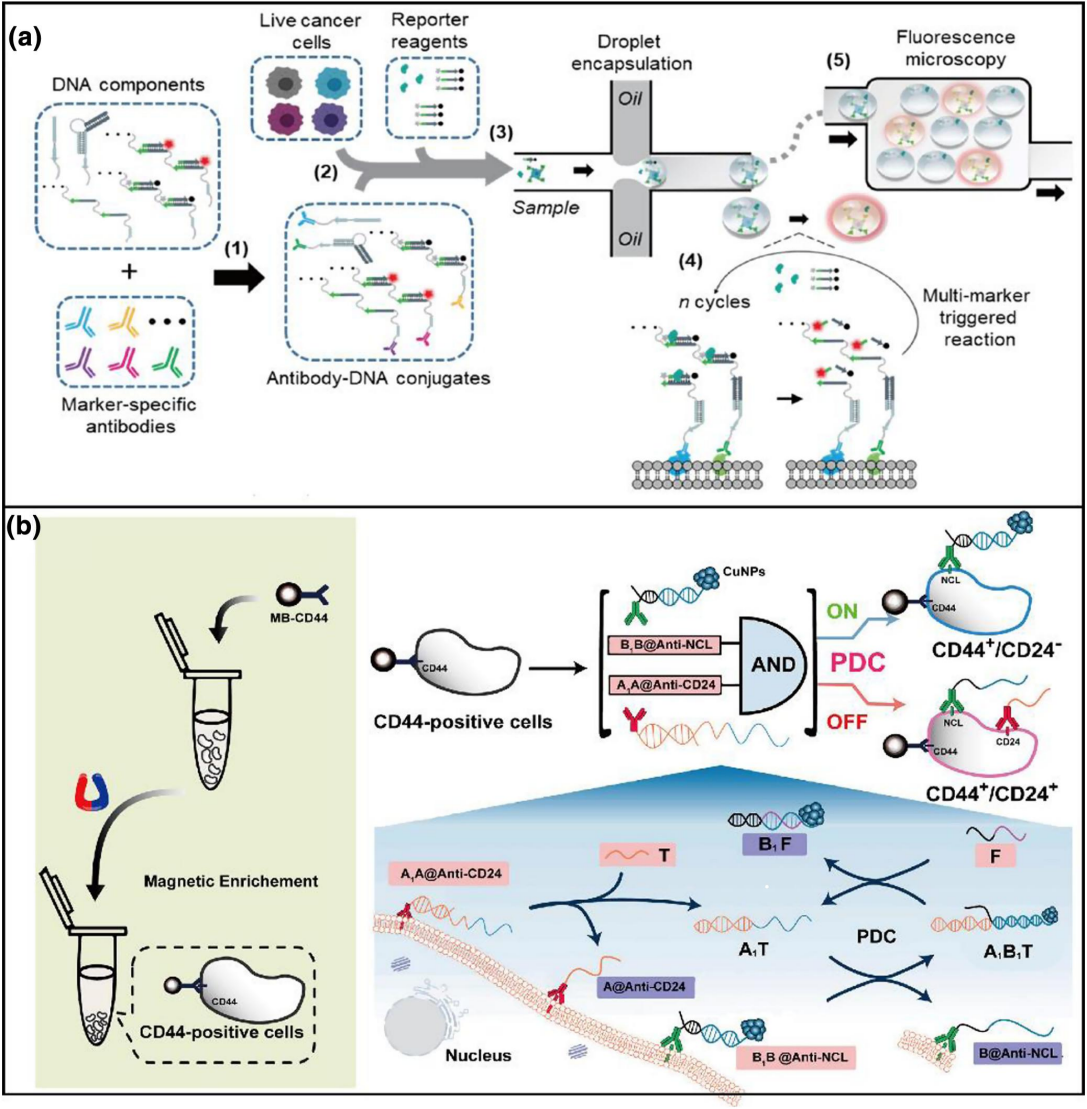
Different signal output methods for the identification of cancer receptors. (a) Overview of fluorescent‐droplet cytometry (FDC).^[38]^ (b) In situ PDC‐based phenotyping method based on electrochemical method.^[40]^ Graph copyright from reference [^38^, ^40^].

In the above examples, benefiting from the advantages of high throughput, precision, and fast speed, flow cytometry (FCM) is the most used technology to determine the heterogeneous characteristics of a single cell depending on fluorescently labeled molecules.[^32^, ^33^, ^34^] To further perform single‐cell analysis for low‐abundance target cells, the droplet microfluidic platform is more attractive. Kelly's group^[38]^ reported a fluorescent drop cytometry (FDC) strategy for live‐cell phenotypic profiling with a small (<40 cells) number of cells (Figure 5a). This method utilized DNA‐functionalized antibodies to target the cells, in which the DNA‐antibody conjugates could generate amplified fluorescence signals in the droplets with the catalytic reaction of a nicking endonuclease. The oil‐aqueous systems of the microdroplets enable the compartmentalization of individual target cells into discrete reaction chambers that enhance mixing and reaction rates. Finally, the drop fluorescence can be read out to achieve living phenotypic profiling of markers on cell membranes. Though the FCM plays an important role in cell analysis and sorting, several hurdles have limited its application, including the inevitable measurement errors, the requirement of expensive instruments, high‐content data analysis, and incorrect compensation, which require extensive experience of researchers.^[39]^ Accordingly, Zhao et al^[40]^ reported an in situ programmable DNA circuit‐based electrochemical method to determine stemlike cells (Figure 5b). Firstly, CD44‐positive (CD44^+^) breast cancer cells are enriched by anti‐CD‐functionalized magnetic beads (MB‐CD44). Then nucleolin (NCL) and CD24 as another two membrane anchors are respectively recognized by two capture probes (A1A@AntiCD24 and B1B@Anti‐NCL). Two effector probes (T and F) are recruited to part act as the toeholds for the DNA strand crossover events. Finally, signal strand B1‐templated copper nanoparticles (CuNPs‐B1) are removed from the interface of CD24^+^ interfering cells. By collecting stripping voltammetry signals of CuNPs with an electrochemical technique, the phenotyping of stemlike phenotype in breast cancer exhibits an “always‐on” electrochemical method.

## 3 CELL BIOMARKER FOR DIFFERENTIATING SUBTYPES OF CANCER BASED ON LIQUID BIOPSY SAMPLE

Over the past several decades, liquid biopsy has received enormous attention. The method analyzes tumors using biomarkers circulating in fluids. It is considered a minimally invasive and repeatable method of clinical implications, though it is not yet applied to clinical experiments as a standard tool.

*Extracellular vesicles for discrimination*. Extracellular vesicles (EVs), composed of a phospholipid bilayer, are secreted by cells carrying abundant biomarkers including nucleic acids (RNA and DNA), proteins, and metabolites.^[41]^ It has been reported that EVs participate in many biological activities such as tumor initiation, progression, and metastasis through short‐range and long‐range effects.[^1^, ^42^] Therefore, precisely quantifying and characterizing cancer exosomes may help to better understand the heterogeneity of cancer, allowing the implementation of more effective and targeted treatments for cancer patients. In the past years, diagnostic techniques based on extracellular vesicles (EVs) have attracted tremendous attention.

**FIGURE 6:**
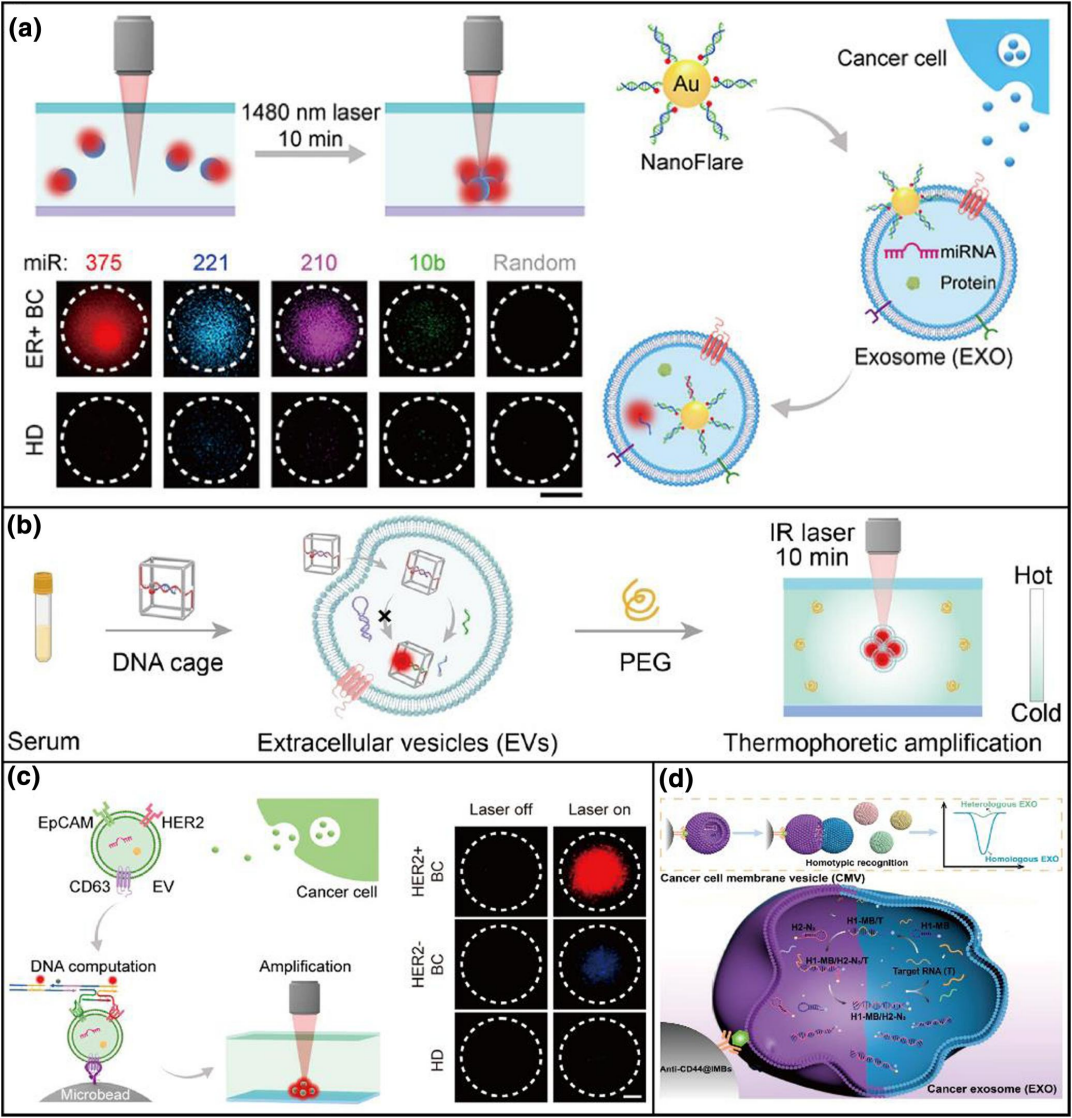
DNA nanosensors based on EVs for discrimination of cancer subtypes. (a) Nanoflares‐based on the thermophoretic sensor for in situ detection of exosomal miRNAs.^[44]^ (b) The thermophoretic implemented with size‐selective DNA cage for detection of mature miRNAs in EVs.^[45]^ (c) Thermophoresis‐mediated DNA computation approach for detection of receptors on EV membranes.^[46]^ (d) Homotypic recognition‐driven molecular characterization of BC exosomes (EXOs) based on using camouflaging catalytic DNA machinery.^[47]^ Graph copyright from reference [^44^, ^45^, ^46^, ^47^].

As a kind of excretion, the EVs contain a set of RNA similar to cancer cells, termed exosomal miRNAs. They potentially mediate paracrine and endocrine communication among different tissues, thus influencing the gene expression and consequential function of distal cells. Dysregulation of the processes can cause tissue dysfunction, aging, and disease. Therefore, these miRNAs are valuable targets for the noninvasive diagnosis of cancer at an early stage. However, the detections of exosomal miRNAs meet formidable challenges due to the low abundance of miRNAs in EXOs. Fortunately, thermophoresis offered an efficient strategy for detecting the EXOs with high sensitivity. Thermophoresis occurs in the presence of a temperature gradient which can be as the transport force to determine particle collection efficiency. Similarly, EXOs could act as a kind of nanoparticle to be significantly enriched in thermophoresis.^[43]^ For example, Sun's group^[44]^ reported thermophoretic nanoflares to detect exosomal miRNAs to discriminate the subtypes of cancer cells (Figure 6a). After incubation with EXOs, the nanoflares can hybridize with the miR‐375 target to generate a fluorescence signal. Then, the EXOs containing nanoflares were enriched by thermophoresis to generate a considerable fluorescent signal. This method achieves an accuracy of 85% in the recognition of estrogen receptor‐positive breast cancer at early stages. Nevertheless, most nanosensors easily degrade in serum and the pre‐miRNAs (longer precursor miRNAs) containing identical sequences in EVs remained an issue to precisely recognize mature miRNAs. To meet these challenges, Sun et al^[45]^ devised DNA cages that have considerable selectivity of size and stability in thermophoresis for the detection of mature miRNAs in EVs (Figure 6b). With this design, the sensor can detect mature miRNAs at a LOD of 2.05 fM without the interference of pre‐miRNAs, which is comparable to the results of gold standard RT‐PCR. This is the first time to detect mature miRNAs in EVs, which improves the detection accuracy in clinical samples and indicates the importance of design in DNA sensors. Except for miRNAs, the receptors on the surface of EVs are also important targets for cancer subtyping. By binding with fluorescent aptamers on the surface of EXO, the surface protein profiles of enriched EXOs can also be measured by thermophoresis technology. For instance, Sun et al^[46]^ reported a strategy of using DNA computation for the molecular phenotyping of breast cancer (BC) (Figure 6c). In their design, Epithelial cell adhesion molecule (EpCAM) and human epidermal growth factor receptor 2 (HER2) expressed on the tumor‐derived extracellular vesicles are selected as biomarkers for the identification of breast cancer (BC) cells associated with this. Two ssDNA strands that target membrane proteins on EV contain three regions: an aptamer recognizing part, a spacer, and an associative toehold activation region. After the recognition of both strands, an HCR takes place for signal amplification. This method finally achieves the discrimination of HER2+ BC, HER2‐BC, and HD in clinical serum samples accurately. These examples exhibit the application potential of thermophoresis for DNA sensors in cancer discrimination.

In addition to thermophoresis, Li et al^[47]^ designed an electrochemical method that contained a biomimetic logical vesicle camouflaging a piece of catalytic DNA machinery with a breast cancer cell membrane (Figure 6d). Cell membranes are camouflaging materials that can facilitate the transport of delivered molecular cargo due to their large encapsulation volume, excellent biocompatibility, low immunogenicity, and stability in the circulatory system. Benefiting from the homotypic recognition of cancer cell membranes, this kind of vesicle can recognize breast cancer (BC) exosomes with similar subtype signatures. The catalytic hairpin assembly of hairpin DNA probes inside the cancer cell membrane (CMV) can be triggered by the inner biomarkers (miR‐375 or PD‐1 mRNA) derived from BC cancer cells. Meanwhile, the CMV of surface‐expressed CD44 overexpressed in BC was immobilized onto anti‐CD44‐functionalized immune‐magnetic beads. The generation of high levels of CHA is enriched on an electrode via click chemistry and subsequent magnetic separation. Then, the amplified electrochemical signals are generated. Different from recognition by aptamer for membrane protein, the method based on homotypic recognition provides a new idea for precise cancer diagnosis.

*Serum samples for discrimination*. It has been reported that non‐coding miRNA expression levels are extremely informative for classifying tumor and normal cells compared to other genomic and proteomic marker molecules, which have become notable tumor markers for cancer diagnosis.^[18]^ Research shows that serum also contains plenty of stable miRNAs that are derived from various tissues/organs. The serum miRNA expression profile can be used as a potential biomarker for early diagnosis of cancer. Despite the progress that DNA sensors have made in cancer subtyping, the clinical diagnostic procedure is still unpleasant and inconvenient. Traditional methods, such as RT‐qPCR, microarrays, and RNA sequencing are cost‐prohibitive, labor‐intensive, and hardware limited under different situations.^[48]^ In addition, individual non‐negligible errors in parallel operations and complex data analysis hinder the development of these methods.

**FIGURE 7:**
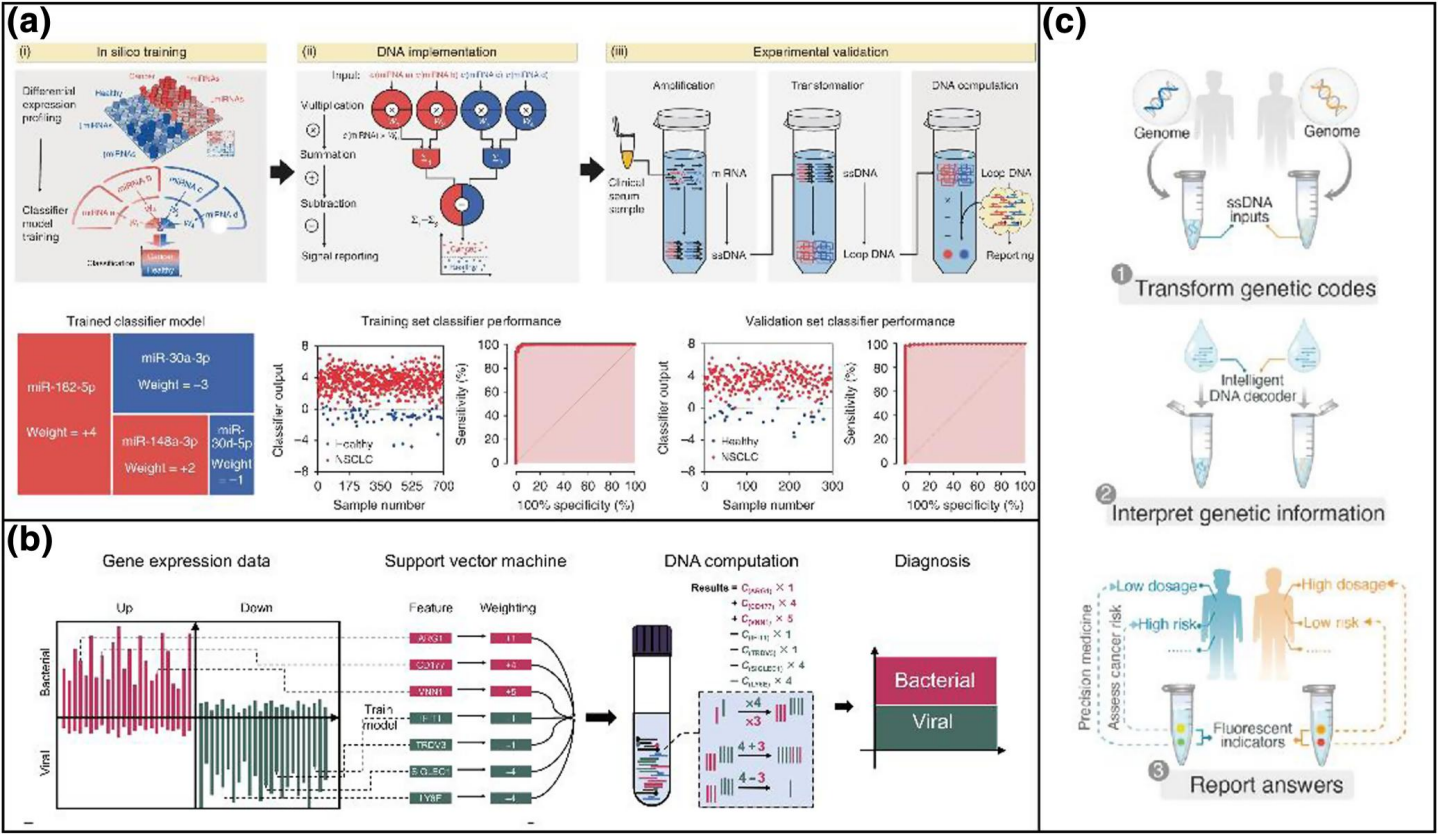
DNA computation platform based on biomarkers in serum samples for discrimination. Workflow for (a) NSCLC diagnosis^[49]^ and (b) ARI etiology^[50]^ with the DNA computation platform. (c) Scheme of an intelligent DNA‐based genetic decoder.^[51]^ Graph copyright from reference [^49^, ^50^, ^51^].

DNA computing has exhibited excellent advantages in logical biosensing. However, it is rarely employed in diagnostic applications, especially in clinical serum samples due to several technical difficulties. First, the incompatibility of biomarker inputs in complex biological systems with simple Boolean values could affect the accuracy of the diagnosis. Second, many specific biomarkers shared with similar sequences impact the orthogonality in the computation. Additionally, a robust and sensitive design is required for the highly dynamic analyte concentration ranges in the sensing of biological samples. Accordingly, Han's group^[49]^ designed a DNA molecular computation platform with a DNA decoder that operates on miRNAs in serum (Figure 7a). This platform includes three main steps: (i) in silico training using publicly available miRNA‐seq profiles of healthy and non‐small cell lung cancer (NSCLC) individuals from TCGA; (ii) DNA implementation of multiplication, summation, and subtraction (iii) and experimental validation on synthetic and clinical serum samples. This DNA‐computing approach achieved NSCLC diagnosis in a 2‐ml serum sample within 6 h with an accuracy of 86.4%, which exhibited unprecedented potential for more clinical applications. On the bias of the approach, they also developed an automated DNA computing‐based platform for acute respiratory infections (ARI) etiology diagnostics and obtained a diagnostic accuracy of 87% in 80 clinical samples without demanding technicians (Figure 7b).^[50]^ Permutation of single‐nucleotide polymorphisms is a kind of variation of nucleic acids, which can lead to a series of phenotypic consequences including human diseases or pathogenic drug resistance. Therefore, they built a programmable DNA decoding circuit to facilitate the translation of complex genetic profiles into actionable medical decisions (Figure 7c). By a ligase‐based signal transforming strategy, multiple allelic information was converted into a series of ssDNA uniquely mapped to DNA gates in the DNA decoding circuit.^[51]^ On the interpenetration of “genetic codes” of CYP2C19, pharmaceutics (PGx) gene, into drug responses, the DNA decoding circuit achieved desired performance with accuracy equivalent to the results of Sanger sequencing in 30 human genomic samples. DNA computing‐based platform combines an in silico classifier trained by publicly available data, a molecular implementation with a DNA‐based reaction network, and a practical workflow with sample amplification, indicating the powerful calculating ability in cancer subtyping.

## 4 CHALLENGES AND OPPORTUNITIES

Despite the progresses that DNA sensors have achieved in the field of cancer subtyping, there are still many challenges that should be overcome to further improve their performance. A general drawback is the poor stability of single‐stranded DNA (ssDNA) in complex biological environments including blood and cytoplasm. The degradation of unmodified DNA biosensors by nucleases may result in a false positive signal, hindering the accuracy of classification. To address this issue, the sensors that classify the subtypes of cancer via intracellular biomarkers can employ various nanomaterials as carriers to reduce the degradation of the sensors as well as improve cellular uptake.[^1^, ^52^] Alternatively, the DNA nanostructures that are completely made up of synthetic DNA strands, such as DNA nanotubes, DNA polyhedrons, and DNA origami possess similar capacities.^[53]^ Additionally, the high programmability and addressability of DNA nanostructures allow the precise and quantitative assembly of DNA sensors, which can perform delicate DNA computations and logical gates either on cell surface or in cells.^[8]^ Apart from the poor stability of DNA mentioned above, another drawback is that the current research is performed in the laboratory. The data results need to be verified by several clinical experiments on unified criteria. Beyond that, a simple upstream design and portable method would be more suitable for clinical application. The above problems have to be considered in the clinical translation of these sensors.

Another promising route that can improve the stability of DNA sensors is the introduction of artificial nucleic acids. For example, the locked nucleic acid (LNA),^[54]^ peptide nucleic acid (PNA),^[55]^ 2′‐fluoroarabinonucleic acids (FANA),^[56]^ (3′‐2′) a‐l‐threose nucleic acid (TNA),^[57]^ and xenonucleic acids (XNA)^[58]^ have been demonstrated to resist the degradation of natural nucleases. When employed as recognition moieties, these artificial nucleic acids still possess the ability to hybridize with natural nucleic acids and can be used for nucleic acid detection to some degree. However, straightforwardly replacing the nucleic acids in current aptamer sequences with artificial nucleic acids may entirely abolish their recognition capability because the artificial nucleic acids hinder the formation of the original second or tertiary structures of aptamers. Recently, Zhu's group^[59]^ attempted to address this issue fundamentally by developing a “mirror‐image selection” strategy, which utilizes mirror‐image DNA polymerase in the processes of systematic evolution of ligands by exponential enrichment (SELEX) to produce L‐DNA aptamers. This kind of aptamer possesses the characteristics of good biostability as well as excellent recognition capability. Once applied to the fabrication of DNA sensors, the L‐DNA aptamers can no doubt accelerate the development of cancer subtyping.

Current DNA sensors often employ fluorescence as signal outputs. Although fluorescent signals have the advantages of high sensitivity and flexible designability, they are susceptible to working environments. The excitation lasers that are required for fluorescent sensors could also cause considerable autofluorescence in complex biological samples, particularly blood, hindering the direct detection of cancer subtypes.^[60]^ Bioluminescent signals that do not require any excitation lights provide promising solutions to this problem. However, integrating the bioluminescent signal outputs into DNA sensors has been a challenge for a long time. Although several attempts have been made to construct bioluminescent DNA sensors, the cumbersome preparation and poor performance limit their applicability in bioassay. Recently, our group^[61]^ reported a universal strategy for the construction of bioluminescent DNA sensors, in which a fusion of Nanoluciferase and Halotag was engineered to bioorthogonally conjugate to DNA sensors. The sensor showed superior performance either in blood detection or in vivo imaging. This general method may provide powerful tools for the detection of cancer subtypes in blood or other biological samples. Another defect of fluorescence is the crosstalk between fluorophores that limits the capacities of DNA sensors in the analysis of multiple targets simultaneously. To address this issue, some high throughput output models, such as mass spectrum^[62]^ and sequencing,^[63]^ might be promising candidates.

As a kind of polyphenotypic disease, clinical diagnosis of cancer subtypes relies on the investigation of heterogeneities in genomics, proteomics, and metabolome simultaneously. Though DNA sensors have been demonstrated to detect a variety of targets, including metal ions, small molecules, proteins, peptides, and cells, current DNA sensors usually focus on a single target category. Therefore, the multi‐dimensional detection of the heterogeneities in different categories of targets may largely improve the accuracy of DNA sensors in cancer subtyping. Additionally, some newly discovered cellular components are also potential biomarkers for cancer subtyping. For example, the glycoRNA which was first demonstrated to exist in cell membranes by Bertozzi's group was then found to be expressed inhomogeneously on normal and cancer cells.[^12^, ^64^] This may provide a possible candidate for cancer subtyping. Recently, as one of the most common types of protein modification, protein glycosylation, which is associated with tumor subtypes, has also attracted extensive attention from the researchers. We believe that these biomarkers would promote the development of DNA sensors in cancer subtyping.

## CONFLICT OF INTEREST STATEMENT

The authors declare no conflicts of interest.

## Data Availability

Data sharing is not applicable to this article as no new data were created or analyzed in this study.
